# Developing dissemination and implementation competencies for training programs

**DOI:** 10.1186/1748-5908-10-S1-A39

**Published:** 2015-08-20

**Authors:** Margaret Padek, Ross Brownson, Enola Proctor, Graham Colditz, Matthew Kreuter, Maureen Dobbins, Anne Sales, Christine Pfund

**Affiliations:** 1 Prevention Research Center in St. Louis, Washington University in St. Louis, 621 N. Skinker Blvd, St. Louis, MO, 63130, USA; 2 Division of Public Health Sciences and Alvin J. Siteman Cancer Center, Washington University School of Medicine, Washington University in St. Louis, 660 South Euclid Ave, St. Louis, MO, 63110, USA; 3 George Warren Brown School of Social Work, Washington University in St. Louis, One Brookings Dr, St. Louis, MO 63130, USA; 4 Health Communication Research Laboratory, Washington University in St. Louis, 700 Rosedale Avenue, Campus Box 1009, St. Louis, MO, 63112-1408, USA; 5 School of Nursing, National Collaborating Centre for Methods and Tools, McMaster University, 175 Longwood Road South, Suite 210a, Hamilton, ON, L8P 0A1, Canada; 6 Center for Clinical Management Research, Veterans Administration Ann Arbor Healthcare System, 400 N. Ingalls Street, Room 4170, Ann Arbor, MI, 48109-5482, USA; 7 Division of Nursing and Business, School of Nursing, University of Michigan, 400 N. Ingalls Street, Room 4170, Ann Arbor, MI, 48109-5482, USA; 8 Institute for Clinical and Translational Research, Wisconsin Center for Education Research, University of Wisconsin-Madison, 4240 Health Sciences Learning Center, 750 Highland Avenue, Madison, WI, 53706, USA

## Introduction

With demand increasing for dissemination and implementation (D&I) training programs in the United States and abroad, more structured, competency-based and tested curricula are needed to guide these training programs. In the first phase of a National Cancer Institute-sponsored D&I training grant (R25CA171994-02), a qualitative exploratory analysis was conducted to establish a new set of D&I competencies to guide these trainings in D&I research.

## Methods

Based upon existing D&I training literature, our leadership team compiled an initial list of competencies. The competency list was then additionally narrowed to forty-three unique competencies following feedback elicited from several other experienced D&I leaders. Three-hundred D&I researchers were then invited via email to complete a card sorting activity in which the list of competencies were sorted into three categories of experience levels. We calculated a mean score for each competency based on their experience level categorization (e.g. score of 1 for beginner). From these mean scores, beginner, intermediate, advanced level tertiles were created for the competencies. The forty-three competencies were then grouped into four broad domains (background & rationale; theory & approaches; design & analysis; practice-based considerations) and sorted based on their experience level score.

## Results

The card-sort achieved a 41% response rate. Eleven competencies fell into the "Beginner" category; twenty-eight into "Intermediate" and only four into "Advanced."

## Discussion

Most competencies were categorized as "Intermediate", which may indicate that the field is still growing and experts remain unsure of what constitutes intermediate vs. advanced competencies. While more work is necessary with these competencies, these results provide a robust beginning for better articulating what is expected from D&I researchers across experience levels. Training developers can use this competency list to formalize future trainings in D&I research, create more evidence-informed curricula and enable overall capacity building and accompanying metrics in the field of D&I training and research.

